# Prostate-specific antigen testing rates in high-risk populations: results from the All of Us Research Program

**DOI:** 10.1007/s10552-023-01807-7

**Published:** 2023-10-25

**Authors:** Faith Morley, Hari S. Iyer, Rulla M. Tamimi, David M. Nanus, Timothy R. Rebbeck, Kevin H. Kensler

**Affiliations:** 1 Department of Population Health Sciences, Weill Cornell Medicine, 402 E 67th Street LA-265, New York, NY 10065 USA; 2 Section of Cancer Epidemiology and Health Outcomes, Rutgers Cancer Institute of New Jersey, New Brunswick, NJ USA; 3 Sandra and Edward Meyer Cancer Center, Weill Cornell Medicine, New York, NY USA; 4 Division of Hematology and Oncology, NewYork-Presbyterian/Weill Cornell Medicine, New York, NY USA; 5 Division of Population Sciences, Dana-Farber Cancer Institute, Boston, MA USA; 6 Department of Epidemiology, Harvard T.H. Chan School of Public Health, Boston, MA USA

**Keywords:** Prostate-specific antigen, Prostate cancer, Early detection of cancer, Cancer screening, Health disparities

## Abstract

**Background:**

Early detection of prostate cancer using prostate-specific antigen (PSA) remains controversial and disparities in the receipt of prostate cancer screening persist in the US. We sought to examine disparities in PSA testing rates among groups with higher prostate cancer risk and differential access to healthcare.

**Methods:**

We identified a cohort of 37,706 males within the All of Us Research Program without a history of prostate cancer between the ages of 40 and 85 at time of enrollment (2017–2021). Incidence rate ratios (IRR) for the number of PSA tests received during follow-up through December 2021 were estimated using age- and multivariable-adjusted negative binomial regression models. PSA testing frequencies in the cohort were compared with population-based estimates from the 2020 Behavioral Risk Factor Surveillance System (BRFSS).

**Results:**

A total of 6,486 males (17.2%) received at least one PSA test over the course of follow-up. In multivariable-adjusted models, non-Hispanic Black males received PSA tests at a 17% lower rate (IRR = 0.83, 95% CI 0.76, 0.90) than non-Hispanic White males. Higher educational attainment, higher annual income, having self-/employer-purchased insurance, having a spouse or domestic partner, and having a family history of prostate cancer were all associated with higher rates of PSA testing. The proportion of males ages 55 to 69 who received a PSA test within two years was lower in All of Us (12.4%, 95% CI 11.8–13.0%) relative to population-based estimates from the BRFSS (35.2%, 95% CI 34.2–36.3%).

**Conclusion:**

Absolute PSA testing rates in All of Us were lower than population-based estimates, but associations with PSA testing in the cohort mirrored previously reported disparities in prostate cancer screening. These findings highlight the importance of addressing barriers to care in order to reduce disparities in cancer screening.

**Supplementary Information:**

The online version contains supplementary material available at 10.1007/s10552-023-01807-7.

## Introduction

Prostate cancer is the most commonly diagnosed cancer and second leading cause of cancer mortality among men in the United States [1]. Major risk factors for prostate cancer include age, family history of prostate cancer, African ancestry, and obesity [2]. The dearth of modifiable risk factors for prostate cancer highlights the importance of early detection in reducing mortality from prostate cancer. Screening for prostate cancer using serum prostate-specific antigen (PSA) concentrations has been fraught with controversy since its introduction in mid-1980s [3]. The U.S. Preventive Services Task Force currently recommends that men ages 55–69 undergo shared decision-making with their provider, stating that the benefits of PSA-based screening may not outweigh the potential harms [4].

Given the controversy surrounding prostate cancer screening using PSA, screening rates have declined over the past decade [5, 6]. A stage shift in prostate cancer and higher incidence of metastatic disease have been observed following this decline in screening [7, 8]. Factors related to healthcare access and utilization have been linked to PSA testing rates, including income, health insurance status, marital status, veteran status, and state of residence among others [9–14]. Disparities in prostate cancer screening by race and ethnicity have also been persistent and are notable given the large variation in prostate cancer incidence and mortality across racial and ethnic populations in the US [5, 13, 15]. The benefit–harm ratio of PSA-based screening may be better among Black men relative to the general US population given increased incidence of aggressive disease among Black men, but evidence regarding these tradeoffs is lacking due to low representation of Black men in large screening trials [16–18]. This study aimed to evaluate the associations between risk factors for prostate cancer as well as factors that influence healthcare access and utilization in the US and PSA testing frequencies, and whether these relationships differed by self-identified race and ethnicity. To address these questions, we leveraged data from a newly established cohort in the U.S. intended to provide health insights for populations traditionally underrepresented in biomedical research.

## Methods

### All of Us Research Program

The All of Us Research Program is a longitudinal cohort initiative funded by the National Institute of Health to create a diverse and expansive dataset including survey data, electronic health record (EHR) data, physical measurements, genomic data, and biospecimens with a concentration on recruiting individuals from populations historically underrepresented in biomedical research [19]. All of Us participants provided written informed consent upon cohort enrollment. For participants who consent to provide EHR data, EHR data are standardized across health provider organizations using the Observational Medial Outcomes Partnership (OMOP) common data model. Data from the All of Us Research Program are publicly available through the All of Us Researcher Workbench (https://workbench.researchallofus.org).

To evaluate patterns in PSA testing, we identified a cohort of participants using the Controlled Tier Version 6 data release who were assigned male at birth, identified as male, were between the ages of 40 and 85 at enrollment, had body mass index (BMI) measurements on record, and who had at least one condition, observation, or procedure within their linked EHR. Males with a history of prostatic disease or prostate cancer at enrollment, unknown self-identified race or ethnicity, who resided in U.S. territories, or who had no follow-up time after enrollment were excluded (Supplemental Fig. 1). For 13 of the 50 health provider organizations contributing EHR data, zero PSA tests were recorded among participants at these sites. Correspondingly, participants enrolling at these 13 sites were excluded, as the lack of any documented PSA tests at these sites may reflect failure to incorporate PSA tests in the standardized EHR data across sites rather than a true absence of testing across all participants at that site. Follow-up for PSA tests was evaluated from time of enrollment (between 2017 and 2021) until death, diagnosis of incident prostatic disease, diagnosis of incident prostate cancer, or end of follow-up on 31 December 2021. To remove the impact of the COVID-19 pandemic on PSA testing rates, a sensitivity analysis was conducted among 30,171 participants who enrolled in 2019 or earlier and that truncated follow-up for PSA tests on 31 December 2019.

### Ascertainment of key variables in All of Us

Participant receipt of a PSA test was ascertained by querying the linked EHR data for eight distinct codes: “19195-7,” “2857-1,” “35741-8,” “377981000000102,” “63476009,” “83112-3,” “84153,” and “G0103.” These included codes for PSA lab results, codes for the fulfillment of the PSA test, or a provider’s order of the PSA test.

Participant history of prostatic disease or prostate cancer at enrollment was ascertained through questionnaire and EHR data. Incident prostate cancer or prostatic disease were identified through EHR records. Participant date of birth, race and ethnicity, state of residence, educational attainment, housing, employment status, income, health insurance status, and relationship status were self-reported in the ‘Basics’ survey. Family history of prostate cancer was determined through the optional ‘Family Health History’ survey. Finally, body mass index (BMI) was calculated based on height and weight measures completed during an in-person study enrollment visit.

### Comparison to Behavioral Risk Factor Surveillance System

To examine PSA testing frequencies in the All of Us Research Program relative to national population-based estimates, the 2020 Behavioral Risk Factor Surveillance Study (BRFSS) national survey results were utilized as a comparator [20]. This state-based phone interview study asks participants if they have received a PSA test in the past two years and is generalizable to the US population. Applying survey weights to the BRFSS, the proportion of men who received a screening PSA test within the past two years was estimated among a subpopulation of participants ages 55–69 who had no history of prostate cancer [5]. The analogous proportion was evaluated in the All of Us Research Program by identifying a cohort of participants that had at least two years of follow-up time in the cohort. Three different cohorts were defined in All of Us as comparators to evaluate the inclusion/exclusion criteria that best replicated screening frequencies observed nationally. Cohort A contained males ages 55–69 who did not have a history of prostate cancer and included all health provider organizations regardless of whether any PSA tests were recorded among participants enrolling at that health provider organization. Cohort B was the primary cohort used in all other analyses and contained males ages 55–69 who did not have a history of prostate cancer or prostatic disease and excluded 13 of the 50 All of Us health provider organizations at which no individuals recorded a PSA test over follow-up in the linked EHR data. Finally, eligibility for Cohort C was the same as for Cohort B while including men with a history of prostatic disease at baseline.

### Statistical analyses

Distributions of participant characteristics among those who had and had not received a PSA test over follow-up were compared using chi-squared tests. To account for overdispersion in the distribution of the number of PSA tests, age- and multivariable-adjusted negative binomial regression models were fit to estimate the incidence rate ratios (IRR) for the frequency of PSA tests. The multivariable-adjusted model accounted for prostate cancer risk factors and factors related to healthcare access and utilization including age at enrollment, BMI, family history of prostate cancer, race and ethnicity, country of birth, Census Division of residence, educational attainment, annual income, employment status, housing, health insurance status, veteran status, and relationship status. Effect modification between race and ethnicity and age at enrollment, country of birth, Census Division of residence, educational attainment, annual income, employment status, housing, health insurance status, relationship status, BMI, and family history of prostate cancer was evaluated using likelihood ratio tests for the inclusion of product terms. Effect modification was only assessed among non-Hispanic Black and non-Hispanic White males, given the limited sample size of males with other self-reported racial or ethnic identities. For all nominal categorical variables, the largest category was considered as the reference group. All analyses were completed using R (version 4.2.2) in the All of Us Research Workbench. To adhere to All of Us Research Program participant confidentiality requirements, all cells with < 20 individuals have been suppressed.

## Results

A total of 37,706 men met eligibility criteria in the All of Us Research Program and were followed for a median 30.3 months (interquartile range: 23.8, 37.5 months), over which 6,486 (17.2%) received at least one PSA test (Table 1). Among males ages 55 to 69 years, 21.6% received at least one PSA test, while the proportions were lower among males ages 40 to 54 years (11.5%), ages 70 to 79 (19.0%), and over 80 years (8.3%). The cohort was predominantly composed of males who self-identified as non-Hispanic White (62.9%) or non-Hispanic Black (30.8%), with lower representation of non-Hispanic Asian or Pacific Islander (2.8%), Hispanic (1.8%), non-Hispanic multiracial (1.2%), and non-Hispanic Middle Eastern or North African males (0.6%). Crude associations with receipt of one or more PSA tests over follow-up were observed for participant race and ethnicity, educational attainment, income, home ownership, health insurance status, relationship status, Census division of residence, BMI, and family history of prostate cancer.

**Table 1:** All of Us Research Program participant characteristics stratified by PSA testing status over follow-up from enrollment (2017–2021) through December 2021

Characteristic^a^	Total^b^ *n* = 37,706	Received 1 + PSA tests^b^ *n* = 6,486	No PSA tests^c^ *n* = 31,220	*p*-value^d^
Year of enrollment				
2017	1,274 (3.4%)	299 (23.5%)	975 (76.5%)	< 0.001
2018	10,811 (28.7%)	2,033 (18.8%)	8,778 (81.2%)	
2019	18,092 (48.0%)	3103 (17.2%)	14,989 (82.8%)	
2020	4,269 (11.3%)	819 (19.2%)	3,450 (80.8%)	
2021	3,260 (8.6%)	232 (7.1%)	3,028 (92.9%)	
Age at enrollment				
40 to 44	3,712 (9.8%)	210 (5.7%)	3,502 (94.3%)	< 0.001
45 to 49	4,507 (12.0%)	475 (10.5%)	4,032 (89.5%)	
50 to 54	5,717 (15.2%)	921 (16.1%)	4,796 (83.9%)	
55 to 59	6,581 (17.5%)	1273 (19.3%)	5,308 (80.7%)	
60 to 64	6,089 (16.1%)	1381 (22.7%)	4,708 (77.3%)	
65 to 69	4,947 (13.1%)	1145 (23.1%)	3,802 (76.9%)	
70 to 74	3,484 (9.2%)	731 (21.0%)	2,753 (79.0%)	
75 to 79	1,846 (4.9%)	282 (15.3%)	1,564 (84.7%)	
80 to 85	823 (2.2%)	68 (8.3%)	755 (91.7%)	
Race and ethnicity				
Hispanic	666 (1.8%)	87 (13.1%)	579 (86.9%)	< 0.001
Non-Hispanic Asian or Pacific Islander	1,055 (2.8%)	198 (18.8%)	857 (81.2%)	
Non-Hispanic Black	11,598 (30.8%)	1,279 (11.0%)	10,319 (89.0%)	
Non-Hispanic Middle Eastern or North African	237 (0.6%)	43 (18.1%)	194 (81.9%)	
Non-Hispanic Multiracial	438 (1.2%)	62 (14.2%)	376 (85.8%)	
Non-Hispanic White	23,712 (62.9%)	4,817 (20.3%)	18,895 (79.7%)	
Country of birth				
USA	34,537 (91.6%)	5,871 (17.0%)	28,666 (83.0%)	< 0.001
Outside USA	2,733 (7.2%)	555 (20.3%)	2,178 (79.7%)	
Unknown	436 (1.2%)	60 (13.8%)	376 (86.2%)	
Census division of residence				
New England	4,730 (12.5%)	1,077 (22.8%)	3,653 (77.2%)	< 0.001
Middle Atlantic	6,125 (16.2%)	1,675 (27.3%)	4,450 (72.7%)	
South Atlantic	3,618 (9.6%)	341 (9.4%)	3,277 (90.6%)	
East South Central	2,864 (7.6%)	324 (11.3%)	2,540 (88.7%)	
East North Central	9,120 (24.2%)	1,716 (18.8%)	7,404 (81.2%)	
West South Central	1,540 (4.1%)	379 (24.6%)	1,161 (75.4%)	
West North Central	780 (2.1%)	280 (35.9%)	500 (64.1%)	
Mountain	5,049 (13.4%)	114 (2.3%)	4,935 (97.7%)	
Pacific	3,880 (10.3%)	580 (14.9%)	3,300 (85.1%)	
Educational attainment				
Less than high school	3,123 (8.3%)	287 (9.2%)	2,836 (90.8%)	< 0.001
High school graduate	8,812 (23.4%)	1,089 (12.4%)	7,723 (87.6%)	
Some college	9,116 (24.2%)	1,509 (16.6%)	7,607 (83.4%)	
College	7,567 (20.1%)	1,613 (21.3%)	5,954 (78.7%)	
Advanced degree	8,002 (21.2%)	1,902 (23.8%)	6,100 (76.2%)	
Unknown	1,086 (2.9%)	86 (7.9%)	1,000 (92.1%)	
Annual income				
Less than $10,000	6,630 (17.6%)	580 (8.7%)	6,050 (91.3%)	< 0.001
$10,000 to $24,999	4,601 (12.2%)	647 (14.1%)	3,954 (85.9%)	
$25,000 to $34,999	2,108 (5.6%)	352 (16.7%)	1,756 (83.3%)	
$35,000 to $49,999	2,341 (6.2%)	448 (19.1%)	1,893 (80.9%)	
$50,000 to $74,999	3,314 (8.8%)	710 (21.4%)	2,604 (78.6%)	
$75,000 to $99,999	2,880 (7.6%)	620 (21.5%)	2,260 (78.5%)	
$100,000 to $149,999	3,854 (10.2%)	941 (24.4%)	2,913 (75.6%)	
$150,000 to $199,999	1,909 (5.1%)	483 (25.3%)	1,426 (74.7%)	
Over $200,000	3,243 (8.6%)	883 (27.2%)	2,360 (72.8%)	
Unknown	6,826 (18.1%)	822 (12.0%)	6,004 (88.0%)	
Employment status				
Employed	14,404 (38.2%)	2,977 (20.7%)	11,427 (79.3%)	< 0.001
Retired	10,242 (27.2%)	2,039 (19.9%)	8,203 (80.1%)	
Unable to work	6,875 (18.2%)	916 (13.3%)	5,959 (86.7%)	
Unemployed/Student/Homemaker	5,166 (13.7%)	461 (8.9%)	4,705 (91.1%)	
Unknown	1,019 (2.7%)	93 (9.1%)	926 (90.9%)	
Housing				
Own	18,333 (48.6%)	4,254 (23.2%)	14,079 (76.8%)	< 0.001
Rent	12,999 (34.5%)	1,668 (12.8%)	11,331 (87.2%)	
Other	4,556 (12.1%)	417 (9.2%)	4,139 (90.8%)	
Unknown	1,818 (4.8%)	147 (8.1%)	1,671 (91.9%)	
Insurance status				
Medicare	8,923 (23.7%)	1,683 (18.9%)	7,240 (81.1%)	< 0.001
Employer/self-purchased	11,306 (30.0%)	2,635 (23.3%)	8,671 (76.7%)	
Medicaid	9,392 (24.9%)	952 (10.1%)	8,440 (89.9%)	
VA/military	3,286 (8.7%)	756 (23.0%)	2,530 (77.0%)	
None	4,799 (12.7%)	460 (9.6%)	4,339 (90.4%)	
Veteran status				
Veteran	7,953 (21.1%)	1,485 (18.7%)	6,468 (81.3%)	< 0.001
Non-veteran	29,106 (77.2%)	4,935 (17.0%)	24,171 (83.0%)	
Unknown	647 (1.7%)	66 (10.2%)	581 (89.8%)	
Relationship status				
Married/domestic partner	19,359 (51.3%)	4,148 (21.4%)	15,211 (78.6%)	< 0.001
Divorced/widowed/separated	9,168 (24.3%)	1,267 (13.8%)	7,901 (86.2%)	
Never married	8,271 (21.9%)	987 (11.9%)	7,284 (88.1%)	
Unknown	908 (2.4%)	84 (9.3%)	824 (90.7%)	
Body mass index (kg/m^2^)				
Less than 25	9,340 (24.8%)	1,213 (13.0%)	8,127 (87.0%)	< 0.001
25 to 30	13,916 (36.9%)	2,511 (18.0%)	11,405 (82.0%)	
30 to 35	8,348 (22.1%)	1,611 (19.3%)	6,737 (80.7%)	
Greater than 35	6,102 (16.2%)	1,151 (18.9%)	4,951 (81.1%)	
Family history of prostate cancer				
Yes	1,104 (2.9%)	406 (36.8%)	698 (63.2%)	< 0.001
No	11,706 (31.0%)	2,893 (24.7%)	8,813 (75.3%)	
Unknown	24,896 (66.0%)	3,187 (12.8%)	21,709 (87.2%)	

^a^Participant body mass index (kg/m^2^) was ascertained during an in-person study visit. All other variables were self-reported in surveys

^b^Displayed percentages are column percentages

^c^Displayed percentages are row percentages

^d^*P*-values are calculated from chi-square test

Many of these associations were similar in multivariable-adjusted negative binomial regression models (Table 2). PSA testing rates increased with age and were 4.65-fold higher among males ages 60 to 65 relative to males 40 to 44 (IRR = 4.65, 95% CI 4.02–5.38), before declining at higher ages. Non-Hispanic Black males had 17% lower rates of PSA testing compared to non-Hispanic White males (IRR = 0.83, 95% CI 0.76–0.90). Relative to non-Hispanic White males, non-Hispanic multiracial males had a 27% lower testing rate (IRR = 0.73, 95% CI 0.55–0.96), while there was no significant difference in the testing rate for Hispanic, non-Hispanic Asian or Pacific Islander, or non-Hispanic Middle Eastern or North African males. Educational attainment and annual income were both positively associated with PSA testing rates, with 22% higher rates among males with an advanced degree compared to males with less than a high school education (IRR = 1.22, 95% CI 1.06–1.42), and a 36% higher rate among males earning $200,000 or more relative to those earning less than $10,000 annually (IRR = 1.36, 95% CI 1.17–1.59). Males who self-reported being unemployed, a student or a homemaker had 20% lower PSA testing rates than those who were employed (IRR = 0.80, 95% CI 0.71–0.90). Relative to individuals with Medicare insurance, individuals with VA or Military healthcare coverage had 1.68-fold higher PSA testing rates (IRR = 1.68, 95% CI 1.49–1.90) and PSA testing rates among men with employer- or self-purchased insurance were modestly higher (IRR = 1.15, 95% CI 1.05–1.27). Relationship status was not associated with PSA testing rates in multivariable-adjusted models. Higher BMI was associated with a higher rate of PSA testing (IRR = 1.34, 95% CI 1.22–1.46, BMI > 35 kg/m^2^ vs. BMI < 25 kg/m^2^). Finally, males with a family history of prostate cancer had a 49% higher PSA testing rate compared to males without (IRR = 1.49, 95% CI 1.31–1.70). Associations were largely similar in magnitude and direction in the analysis truncating follow-up on 31 December 2019, to remove the effect of the COVID-19 pandemic (Supplemental Table 1), although associations between some factors, such as educational attainment, housing, race and ethnicity, and health insurance status, and PSA testing rates attenuated and were no longer statistically significant in this subpopulation with shorter follow-up.

**Table 2:** Age- and multivariable (MV)-adjusted incidence rate ratios (IRRs) and 95% confidence intervals (95% CIs) of PSA testing rates in the All of Us Research Program

Covariates^a^	Age-adjusted IRR	95% CI	*p*-value^b^	MV-adjusted IRR^b^	95% CI	*p*-value^c^
Year of enrollment						
2017	Ref		< 0.001	Ref		< 0.001
2018	0.89	0.77, 1.03		1.06	0.93, 1.22	
2019	0.94	0.82, 1.08		1.12	0.98, 1.28	
2020	1.22	1.04, 1.43		1.33	1.14, 1.55	
2021	1.41	1.15, 1.73		1.31	1.08, 1.59	
Age at enrollment						
40 to 44	Ref		< 0.001	Ref		< 0.001
45 to 49	1.87	1.59, 2.19		1.91	1.63, 2.24	
50 to 54	3.33	2.87, 3.87		3.33	2.87, 3.86	
55 to 59	4.31	3.73, 4.99		4.19	3.63, 4.85	
60 to 64	5.50	4.76, 6.37		4.65	4.02, 5.38	
65 to 69	5.89	5.08, 6.84		4.48	3.82, 5.27	
70 to 74	5.80	4.96, 6.80		3.93	3.31, 4.68	
75 to 79	3.88	3.23, 4.67		2.87	2.36, 3.49	
80 to 85	2.12	1.62, 2.76		1.69	1.28, 2.21	
Race and ethnicity						
Non-Hispanic White	Ref		< 0.001	Ref		< 0.001
Hispanic	0.69	0.55, 0.87		0.96	0.76, 1.21	
Non-Hispanic Asian or Pacific Islander	1.05	0.89, 1.25		1.00	0.84, 1.19	
Non-Hispanic Black	0.46	0.43, 0.49		0.83	0.76, 0.90	
Non-Hispanic Middle Eastern or North African	1.09	0.77, 1.55		1.13	0.79, 1.60	
Non-Hispanic Multiracial	0.66	0.49, 0.87		0.73	0.55, 0.96	
Country of birth						
USA	Ref		< 0.001	Ref		< 0.001
Outside USA	1.36	1.22, 1.51		1.24	1.11, 1.39	
Unknown	0.69	0.53, 0.91		0.92	0.71, 1.19	
Census division of residence						
New England	Ref		< 0.001	Ref		< 0.001
Middle Atlantic	1.22	1.11, 1.34		1.26	1.15, 1.38	
South Atlantic	0.41	0.36, 0.46		0.60	0.53, 0.69	
East South Central	0.43	0.38, 0.49		0.67	0.58, 0.77	
East North Central	0.92	0.85, 1.01		0.99	0.91, 1.08	
West South Central	1.06	0.93, 1.22		1.11	0.97, 1.27	
West North Central	2.07	1.75, 2.46		1.46	1.22, 1.74	
Mountain	0.10	0.09, 0.12		0.12	0.10, 0.14	
Pacific	0.63	0.56, 0.70		0.57	0.51, 0.63	
Educational attainment						
Less than high school	Ref		< 0.001			0.013
High school graduate	1.56	1.37, 1.79		1.13	0.99, 1.29	
Some college	2.25	1.97, 2.57		1.19	1.04, 1.36	
College	2.91	2.55, 3.33		1.17	1.01, 1.35	
Advanced degree	3.42	2.99, 3.90		1.22	1.06, 1.42	
Unknown	0.96	0.76, 1.22		0.88	0.69, 1.11	
Annual income						
Less than $10,000	Ref		< 0.001	Ref		< 0.001
$10,000 to $24,999	1.67	1.49, 1.88		1.17	1.04, 1.32	
$25,000 to $34,999	2.21	1.92, 2.55		1.27	1.09, 1.48	
$35,000 to $49,999	2.62	2.29, 3.00		1.23	1.06, 1.43	
$50,000 to $74,999	3.15	2.80, 3.55		1.30	1.13, 1.49	
$75,000 to $99,999	2.98	2.63, 3.37		1.17	1.01, 1.36	
$100,000 to $149,999	3.47	3.10, 3.89		1.25	1.08, 1.45	
$150,000 to $199,999	3.82	3.33, 4.38		1.35	1.15, 1.60	
Over $200,000	4.02	3.58, 4.51		1.36	1.17, 1.59	
Unknown	1.55	1.39, 1.73		1.05	0.93, 1.18	
Employment status						
Employed	Ref		< 0.001	Ref		0.001
Retired	0.83	0.76, 0.90		0.97	0.89, 1.05	
Unable	0.50	0.46, 0.54		0.99	0.90, 1.10	
Unemployed/student/homemaker	0.38	0.34, 0.42		0.80	0.71, 0.90	
Unknown	0.38	0.31, 0.47		0.88	0.70, 1.09	
Housing						
Own	Ref		< 0.001	Ref		< 0.001
Rent	0.50	0.47, 0.53		0.83	0.77, 0.90	
Other	0.33	0.30, 0.37		0.75	0.66, 0.84	
Unknown	0.30	0.26, 0.36		0.70	0.59, 0.83	
Insurance status						
Medicare	Ref		< 0.001	Ref		< 0.001
Employer/self-purchased	1.67	1.53, 1.83		1.15	1.05, 1.27	
Medicaid	0.59	0.53, 0.65		0.92	0.83, 1.02	
VA/military	1.54	1.39, 1.70		1.68	1.49, 1.90	
None	0.59	0.52, 0.66		1.01	0.89, 1.15	
Veteran status						
Not a veteran	Ref		< 0.001	Ref		0.09
Veteran	1.11	1.04, 1.20		1.03	0.95, 1.12	
Unknown	0.56	0.44, 0.72		0.78	0.61, 0.99	
Relationship status						
Married/domestic partner	Ref		< 0.001	Ref		0.20
Divorced/widowed/separated	0.56	0.52, 0.60		0.95	0.88, 1.03	
Never married	0.51	0.47, 0.55		0.92	0.85, 1.01	
Unknown	0.39	0.31, 0.48		0.83	0.66, 1.03	
Body mass index (kg/m^2^)						
Less than 25	Ref		< 0.001	Ref		< 0.001
25 to 30	1.39	1.29, 1.50		1.18	1.10, 1.27	
30 to 35	1.45	1.33, 1.57		1.23	1.13, 1.33	
Greater than 35	1.50	1.37, 1.64		1.34	1.22, 1.46	
Family history of prostate cancer						
No	Ref		< 0.001	Ref		< 0.001
Yes	1.61	1.40, 1.86	NA	1.49	1.31, 1.70	
Unknown	0.49	0.46, 0.52	NA	0.70	0.66, 0.75	

^a^Participant body mass index (kg/m^2^) was ascertained during an in-person study visit. All other variables were self-reported in surveys

^b^*p*-values are calculated from likelihood ratio test

^c^Multivariable model contains all listed covariates

**Fig. 1:**
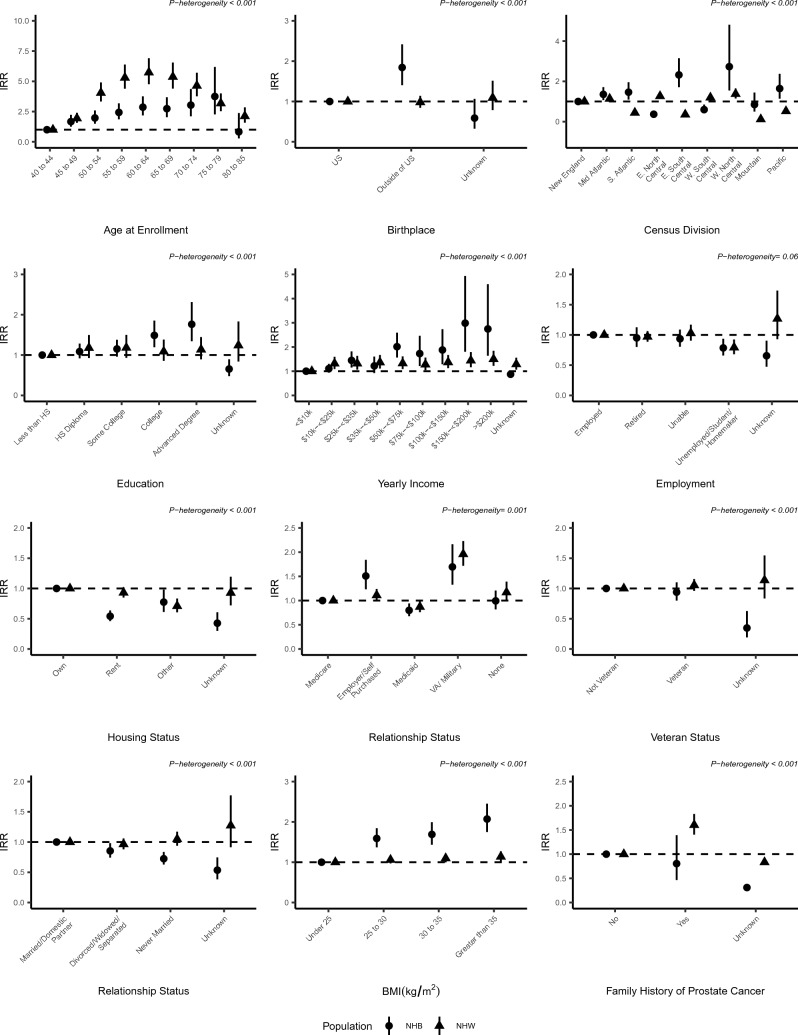
Multivariable-adjusted incidence rate ratios (IRRs) and 95% confidence intervals of PSA testing among non-Hispanic Black (NHB) and non-Hispanic White (NHW) males in the All of Us Research Program. P-heterogeneity is from a likelihood ratio test for the inclusion of a product term between the variable of interest and race and ethnicity

Several factors demonstrated differential associations with PSA testing rates among non-Hispanic Black and non-Hispanic White males (Fig. 1). The association between age at enrollment and PSA testing rates was greater in magnitude among non-Hispanic White males than non-Hispanic Black males (p-heterogeneity < 0.001), particularly in the age group between 55 and 69 years that are recommended to undergo shared decision-making regarding screening. In contrast, educational attainment and income were both positively associated with PSA testing rates among non-Hispanic Black and non-Hispanic White males, however, the magnitude of the association was greater among non-Hispanic Black males (both p-heterogeneity < 0.001). Similarly, the association between BMI and PSA testing rate was greater in magnitude among non-Hispanic Black males (IRR = 2.07, 95% CI 1.75–2.45 comparing > 35 kg/m^2^ to < 25 kg/m^2^) than non-Hispanic White males (IRR = 1.14, 95% CI 1.02–1.27, p-heterogeneity < 0.001). A family history of prostate cancer was associated with higher rates of PSA testing among non-Hispanic White males (IRR = 1.60, 95% CI 1.40–1.83), while there was no association among non-Hispanic Black males (IRR = 0.80, 95% CI 0.46–1.39; p-heterogeneity < 0.001). Non-Hispanic Black males who were never married had lower PSA testing rates relative to those who were married or had a domestic partner (IRR = 0.72, 95% CI 0.63–0.84), while there was no association among non-Hispanic White males (IRR = 1.05, 95% CI 0.94–1.17, p-heterogeneity < 0.001). Non-Hispanic Black males with self- or employer-purchased health insurance had higher PSA testing rates (IRR = 1.48, 95% CI 1.21–1.81) than those with Medicare, although there was parity in testing rates between these groups among non-Hispanic White males (IRR = 1.09, 95% 0.98–1.21; p-heterogeneity = 0.001). Finally, there was no significant heterogeneity in the association between employment status and PSA testing among non-Hispanic Black and non-Hispanic White males.

**Fig. 2:**
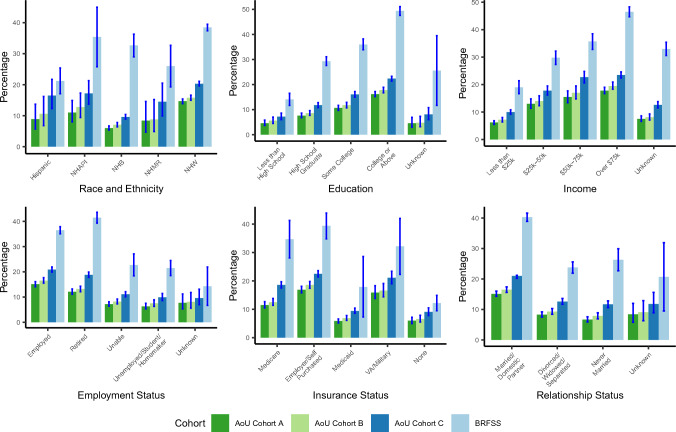
Proportion of males ages 55–69 receiving a PSA test in a two-year period in the 2020 Behavioral Risk Factor Surveillance System (BRFSS) Survey and All of Us (AoU) Research Program. All of Us Research Program data were used to identify three cohorts: **A** Males ages 55–69 without a history of prostate cancer, **B** Males ages 55–69 without a history of prostate cancer excluding EHR sites where no PSA tests were recorded, and **C** Males ages 55–69 without a history of prostate cancer excluding EHR sites where no PSA tests were recorded and including men with prevalent or a history of prostatic disease at study baseline. *NHAPI* non-hispanic asian or pacific islander, *NHB* non-hispanic black, *NHMR* non-hispanic multiracial, *NHW* non-hispanic white

**Fig. 3:**
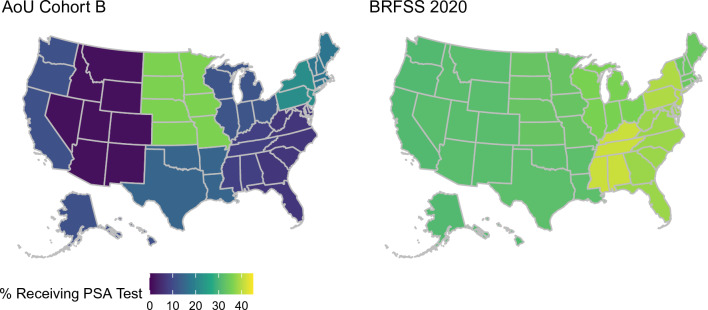
Proportion of males ages 55–69 without a history of prostate care who received a PSA test within a two-year period in the 2020 Behavioral Risk Factor Surveillance System (BRFSS) Survey and All of Us (AoU) Research Program by Census Division. EHR sites where no PSA tests were recorded were excluded in All of Us

PSA testing frequencies from the All of Us Research Program were compared against national population-based estimates from the 2020 BRFSS (Fig. 2). In three subcohorts defined within the All of Us Research Program, the proportion of males ages 55–69 without a history of prostate cancer who received at least one PSA test in the first two years following cohort enrollment was lower (Cohort A: 11.1% [95% CI 10.6–11.6%], Cohort B: 12.4% [95% CI 11.8–13.0%], Cohort C: 16.8% [95% CI 16.2–17.3%]) than the proportion of respondents in the 2020 BRFSS survey who self-reported receiving a PSA test in the past two years (35.2%, 95% CI 34.2–36.3%). Although the absolute proportions of participants receiving PSA tests were considerably lower in All of Us, the All of Us data largely recapitulated the associations between factors influencing healthcare access and utilization, such as educational attainment, income, and PSA testing frequency observed in the BRFSS. There were some notable exceptions. Non-Hispanic multiracial males had the lowest PSA testing frequency across racial and ethnic groups in the BRFSS, while they had higher testing frequencies than non-Hispanic Black males in All of Us. The frequency of PSA testing was higher among retired males relative to employed males in the national BRFSS estimates, while the testing frequencies were higher for employed males relative to retired males in All of Us. Some of the most pronounced differences between All of Us and BRFSS estimates were observed for Census Division of residence (Fig. 3). While BRFSS estimates indicated some modest national variation in PSA testing, with frequencies ranging from 30.9% (95% CI 26.5–35.2%) in the Pacific division, to 41.2% (95% CI 38.3–44.1%) in the East South Central, the magnitude of geographic variation was more substantial in the All of Us Research Program. Moreover, geographic patterns in the All of Us Research Program did not reflect the patterns observed in the population-based estimates from the BRFSS.

## Discussion

We evaluated whether prostate cancer risk factors and factors related to healthcare access and utilization are associated with PSA testing rates in the All of Us Research Program. Our findings largely mirrored the relative measures of association reported in other study populations between these factors and PSA testing frequency in both direction and magnitude. However, absolute PSA testing rates were lower in All of Us in comparison to national cancer surveillance estimates. This stands in contrast to expectations that All of Us participants may be more likely to engage in cancer screening due to healthy volunteer biases [21]. Our findings both reinforce the utility of this new cohort to provide insights into the multilevel drivers of cancer health disparities and highlight potential limitations for leveraging the cohort in future studies of cancer screening and cancer etiology.

In this cohort enriched with participants historically underrepresented in biomedical research, we found that consensus risk factors for prostate cancer such as age, family history of prostate cancer, and obesity were all associated with increased PSA testing. Corresponding with national screening guidelines, PSA testing rates in All of Us were highest among males ages 55–69, with lower rates among men both younger and older than this age range. This U-shaped pattern has been previously observed in national population-based datasets, such as the BRFSS [9, 22]. Though less common than among males ages 55–69 (21.6%), we observed that 13.1% of males ages 75 or older received a PSA test. There is little evidence of a mortality benefit of prostate cancer screening in this age group, and thus these males may be facing a higher risk of overdiagnosis and overtreatment without any potential mortality benefit [11, 23–25].

Notably, we observed a large disparity in PSA testing frequency by participant self-identified race and ethnicity, wherein non-Hispanic Black men and non-Hispanic multiracial men were less likely to receive PSA tests than non-Hispanic White men. However, the magnitude of the disparity between non-Hispanic Black and non-Hispanic White men in All of Us exceeded estimates from population-based surveys [5]. National estimates also suggest that non-Hispanic Black men ages 40–54 are more likely to be screened for prostate cancer than non-Hispanic White men in this age group, though the opposite association was observed in All of Us [26]. This suggests there may be under-ascertainment of PSA tests within the All of Us data, particularly among minoritized racial and ethnic populations. Factors that influence healthcare access and utilization in the US, such as educational attainment, income, housing, and relationship status, were independently linked to PSA testing rates in All of Us and likewise showed heterogeneity in their associations by race and ethnicity. Notably, the magnitude of the associations between these factors and PSA testing rates were stronger among non-Hispanic Black men than non-Hispanic White men. This finding emphasizes that eliminating barriers to healthcare is necessary to reduce cancer screening disparities across racial and ethnic populations.

Population-based studies suggest that there are regional differences in prostate cancer screening [10]. Our analysis of the 2020 BRFSS found that 2-year PSA testing frequencies ranged from 30.9% (Pacific) to 41.2% (East South Central) across regions. The All of Us cohort findings likewise found strong variability across regions (range: 1.4% in Mountain to 36.1% in West North Central), but the geographic trends in All of Us did not correlate with trends from national population-based datasets. Thus, is it likely that the variation in All of Us does not reflect true geographic variability, but rather is a product of under-ascertainment of PSA tests in the standardized EHR data. EHR data are currently available from All of Us participants who enroll through a health provider organization and authorize the sharing of their EHR data. These health provider organizations include regional medical centers, federally qualified health centers, and the Veteran’s Administration [19]. EHR data are then standardized across health provider organizations using a common data model [19]. Under-ascertainment of cancer screening procedures, such as PSA tests, can therefore arise through multiple mechanisms. Many All of Us participants may receive healthcare at other sites than the healthcare provider organization at which they enrolled, and these procedures would not be captured in their EHR record that is ultimately included in the common data repository. Alternatively, there may be data loss in the standardization of EHR data to the common data model. Conderino and colleagues found that the relative prevalence of cancer screening procedures was 46% to 77% lower in a common data model when comparing to the prevalence of these procedures in raw EHR data at a single contributing site [27]. The extent of missingness likely varies across health provider organizations, and given that there are differences in the demographics of patients receiving care across these organizations, there may be differential measurement error leading to over- or underestimation of the associations of interest.

Our study has some notable limitations. First, we were unable to ascertain the precise clinical indication for PSA tests received. However, participants with a history of prostate cancer or other prostatic diseases were excluded in order to restrict to a population that is likely receiving routine PSA tests to screen for prostate cancer. Second, as mentioned above, under-ascertainment of PSA tests was likely differential across health provider organizations and thus likely differed by participant demographics. If the extent of under-ascertainment were greater among more populations experiencing greater socioeconomic disadvantage, this would potentially lead to overestimates of disparities in PSA testing. Part of the follow-up period for this study coincides with the COVID-19 pandemic during which many individuals deferred routine healthcare. However, associations observed in a sensitivity analysis truncating follow-up before the onset of the pandemic largely corresponded to those observed over the entire follow-up period. Our study is strengthened through the diversity of the participants of the All of Us Research Program, which include demographic groups historically underrepresented in biomedical research. In contrast to large national surveys, we are able to leverage EHR data to assess receipt of a PSA test, and do not need to rely on self-report of PSA testing, for which there is lower accuracy than self-reporting of other cancer screening procedures [28].

In total, we observed that absolute PSA testing frequencies were lower in the All of Us Research Program than estimates from national population-based surveys. However, the associations between prostate cancer risk factors and factors influencing healthcare access and utilization and PSA testing largely recapitulated associations reported nationally. These findings highlight the importance of addressing barriers to care in order to ameliorate disparities in cancer screening among marginalized populations.

### Supplementary Information

Below is the link to the electronic supplementary material.

Supplementary file1 (DOCX 87 KB)

## Data Availability

Data used in this study from the All of Us Research Program are available to registered researchers through the All of Us researcher workbench at https://www.researchallofus.org. Data from the Behavioral Risk Factor Surveillance System are made publicly available by the Centers for Disease Control and Prevention at https://www.cdc.gov/brfss.
