# Supplementary material for: Prostate-specific antigen testing rates in high-risk populations: results from the All of Us Research Program
Source: Cancer Causes Control. 2023 Oct 25;35(3):509–21. doi: 10.1007/s10552-023-01807-7 (PMC10838840; doi:10.1007/s10552-023-01807-7)
Supplement: Supplementary file 1 — Supplementary file1 (DOCX 87 KB) [file 10552_2023_1807_MOESM1_ESM.docx]

**Supplemental Figure 1.** Flowchart for identification of analytic population within All of Us Research Program.


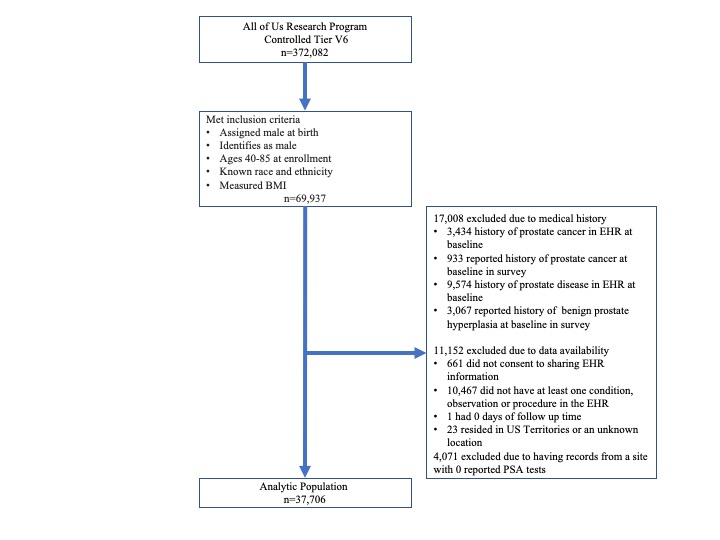


**Supplemental Table 1.** Age- and multivariable (MV)-adjusted incidence rate ratios (IRRs) and 95% confidence intervals (95% CIs) of PSA testing rates with follow-up truncated on December 31, 2019.

| **Covariate** | **Age-Adjusted IRR** | **95% CI** | **P-value^a^** | **MV-Adjusted**  **IRR^b^** | **95% CI** | **P-value^a^** |
| --- | --- | --- | --- | --- | --- | --- |
| **Year of Enrollment** |  |  | 0.004 |  |  | <0.001 |
| 2017 | Ref. |  |  | Ref. |  |  |
| 2018 | 0.93 | 0.81, 1.07 |  | 1.12 | 0.97, 1.28 |  |
| 2019 | 1.07 | 0.92, 1.23 |  | 1.26 | 1.10, 1.45 |  |
| **Age at Enrollment** |  |  | <0.001 |  |  | <0.001 |
| 40 to 44 | Ref. |  |  | Ref. |  |  |
| 45 to 49 | 1.62 | 1.26, 2.09 |  | 1.67 | 1.30, 2.16 |  |
| 50 to 54 | 3.04 | 2.42, 3.85 |  | 3.22 | 2.57, 4.07 |  |
| 55 to 59 | 4.02 | 3.23, 5.06 |  | 4.14 | 3.32, 5.20 |  |
| 60 to 64 | 4.95 | 3.98, 6.23 |  | 4.36 | 3.50, 5.48 |  |
| 65 to 69 | 5.56 | 4.46, 7.02 |  | 4.66 | 3.67, 5.96 |  |
| 70 to 74 | 5.01 | 3.97, 6.39 |  | 3.92 | 3.04, 5.10 |  |
| 75 to 79 | 3.50 | 2.66, 4.62 |  | 2.83 | 2.11, 3.81 |  |
| 80 to 85 | 1.78 | 1.15, 2.69 |  | 1.59 | 1.02, 2.42 |  |
| **Race and Ethnicity** |  |  | <0.001 |  |  | 0.20 |
| Non-Hispanic White | Ref. |  |  | Ref. |  |  |
| Hispanic | 0.70 | 0.50, 0.95 |  | 0.90 | 0.65, 1.23 |  |
| Non-Hispanic Asian Pacific or Islander | 1.22 | 0.98, 1.51 |  | 1.09 | 0.86, 1.37 |  |
| Non-Hispanic Black | 0.56 | 0.51, 0.62 |  | 0.91 | 0.81, 1.02 |  |
| Non-Hispanic Middle Eastern or North African | 0.84 | 0.50, 1.35 |  | 0.77 | 0.46, 1.24 |  |
| Non-Hispanic Multiracial | 0.67 | 0.44, 0.98 |  | 0.74 | 0.49, 1.07 |  |
| **Country of Birth** |  |  | <0.001 |  |  | 0.01 |
| USA | Ref. |  |  | Ref. |  |  |
| Outside USA | 1.43 | 1.25, 1.64 |  | 1.26 | 1.08, 1.46 |  |
| Unknown | 0.70 | 0.48, 0.99 |  | 0.87 | 0.60, 1.22 |  |
| **Census Division of Residence** |  |  | <0.001 |  |  | <0.001 |
| New England | Ref. |  |  | Ref. |  |  |
| Middle Atlantic | 1.32 | 1.17, 1.48 |  | 1.25 | 1.11, 1.40 |  |
| South Atlantic | 0.40 | 0.32, 0.49 |  | 0.53 | 0.43, 0.66 |  |
| East South Central | 0.68 | 0.56, 0.81 |  | 0.87 | 0.71, 1.05 |  |
| East North Central | 0.95 | 0.84, 1.07 |  | 1.01 | 0.89, 1.14 |  |
| West South Central | 1.14 | 0.96, 1.35 |  | 1.10 | 0.92, 1.30 |  |
| West North Central | 2.37 | 1.83, 3.04 |  | 2.15 | 1.63, 2.81 |  |
| Mountain | 0.06 | 0.04, 0.08 |  | 0.07 | 0.05, 0.09 |  |
| Pacific | 0.66 | 0.56, 0.78 |  | 0.58 | 0.50, 0.69 |  |
| **Educational Attainment** |  |  | <0.001 |  |  | 0.15 |
| Less than High School | Ref. |  |  |  |  |  |
| High School Graduate | 1.36 | 1.12, 1.65 |  | 1.09 | 0.90, 1.33 |  |
| Some College | 1.71 | 1.41, 2.07 |  | 1.09 | 0.90, 1.33 |  |
| College | 2.21 | 1.83, 2.68 |  | 1.00 | 0.82, 1.24 |  |
| Advanced Degree | 2.85 | 2.38, 3.45 |  | 1.12 | 0.91, 1.39 |  |
| Unknown | 0.87 | 0.60, 1.22 |  | 0.84 | 0.58, 1.18 |  |
| **Annual Income** |  |  | <0.001 |  |  | 0.01 |
| Less than $10,000 | Ref. |  |  | Ref. |  |  |
| $10,000 to $24,999 | 1.49 | 1.25, 1.76 |  | 1.18 | 0.99, 1.40 |  |
| $25,000 to $34,999 | 1.81 | 1.47, 2.22 |  | 1.19 | 0.96, 1.48 |  |
| $35,000 to $49,999 | 2.07 | 1.70, 2.52 |  | 1.15 | 0.93, 1.43 |  |
| $50,000 to $74,999 | 2.72 | 2.30, 3.23 |  | 1.37 | 1.12, 1.68 |  |
| $75,000 to $99,999 | 2.59 | 2.17, 3.08 |  | 1.25 | 1.01, 1.55 |  |
| $100,000 to $149,999 | 3.08 | 2.63, 3.62 |  | 1.38 | 1.12, 1.70 |  |
| $150,000 to $199,999 | 3.34 | 2.77, 4.03 |  | 1.44 | 1.14, 1.82 |  |
| Over $200,000 | 3.80 | 3.24, 4.45 |  | 1.55 | 1.25, 1.94 |  |
| Unknown | 1.55 | 1.32, 1.81 |  | 1.13 | 0.95, 1.34 |  |
| **Employment Status** |  |  | <0.001 |  |  | 0.07 |
| Employed | Ref. |  |  | Ref. |  |  |
| Retired | 0.80 | 0.72, 0.89 |  | 0.99 | 0.89, 1.11 |  |
| Unable | 0.50 | 0.44, 0.56 |  | 0.93 | 0.80, 1.07 |  |
| Unemployed/Student/Homemaker | 0.40 | 0.34, 0.46 |  | 0.79 | 0.67, 0.94 |  |
| Unknown | 0.39 | 0.28, 0.54 |  | 0.79 | 0.55, 1.10 |  |
| **Housing** |  |  | <0.001 |  |  | 0.05 |
| Own | Ref. |  |  | Ref. |  |  |
| Rent | 0.53 | 0.49, 0.58 |  | 0.91 | 0.82, 1.02 |  |
| Other | 0.35 | 0.29, 0.41 |  | 0.79 | 0.66, 0.94 |  |
| Unknown | 0.36 | 0.28, 0.45 |  | 0.83 | 0.64, 1.06 |  |
| **Insurance Status** |  |  | <0.001 |  |  | 0.07 |
| Medicare | Ref. |  |  | Ref. |  |  |
| Employer/Self Purchased | 1.70 | 1.52, 1.92 |  | 1.17 | 1.02, 1.33 |  |
| Medicaid | 0.65 | 0.56, 0.74 |  | 0.95 | 0.82, 1.11 |  |
| VA/Military | 1.04 | 0.88, 1.21 |  | 1.10 | 0.92, 1.32 |  |
| None | 0.67 | 0.56, 0.80 |  | 1.02 | 0.85, 1.22 |  |
| **Veteran Status** |  |  | <0.001 |  |  | 0.40 |
| Not a Veteran | Ref. |  |  | Ref. |  |  |
| Veteran | 0.89 | 0.80, 0.99 |  | 0.94 | 0.84, 1.06 |  |
| Unknown | 0.55 | 0.38, 0.79 |  | 0.83 | 0.57, 1.18 |  |
| **Relationship Status** |  |  | <0.001 |  |  | 0.01 |
| Married/Domestic Partner | Ref. |  |  | Ref. |  |  |
| Divorced/Widowed/Separated | 0.57 | 0.52, 0.63 |  | 0.94 | 0.84, 1.05 |  |
| Never Married | 0.51 | 0.46, 0.57 |  | 0.88 | 0.78, 1.00 |  |
| Unknown | 0.30 | 0.20, 0.44 |  | 0.59 | 0.39, 0.84 |  |
| **Body Mass Index (kg/m^2^)** |  |  | <0.001 |  |  | <0.001 |
| Less than 25 | Ref. |  |  | Ref. |  |  |
| 25 to 30 | 1.36 | 1.23, 1.51 |  | 1.16 | 1.05, 1.29 |  |
| 30 to 35 | 1.35 | 1.20, 1.52 |  | 1.17 | 1.05, 1.31 |  |
| Greater than 35 | 1.42 | 1.25, 1.61 |  | 1.29 | 1.14, 1.46 |  |
| **Family History of Prostate Cancer** |  |  | <0.001 |  |  | <0.001 |
| No | Ref. |  |  | Ref. |  |  |
| Yes | 1.60 | 1.34, 1.90 |  | 1.47 | 1.25, 1.73 |  |
| Unknown | 0.53 | 0.49, 0.58 |  | 0.78 | 0.72, 0.85 |  |

^a^ P-values are calculated from likelihood ratio test

^b^ Multivariable model contains all listed covariates
